# Dry Beriberi and Acute Motor-Sensory Axonal Neuropathy-Induced Paralysis: A Case Report

**DOI:** 10.7759/cureus.85276

**Published:** 2025-06-03

**Authors:** Marcelo Salazar, Awab Elnaeem, Xiang Fang

**Affiliations:** 1 Neurology, University of Texas Medical Branch, Galveston, USA

**Keywords:** axonal neuropathies, electromyography, guillain-barré syndrome variants, nerve conduction studies, thiamine deficiency

## Abstract

Acute motor-sensory axonal neuropathy (AMSAN) is a variant of Guillain-Barré syndrome (GBS) and a rare cause of paralysis. AMSAN can present similarly to axonal neuropathy caused by thiamine (vitamin B1) deficiency, which is known as dry beriberi. Subtle differences in clinical presentation and diagnostic differences in nerve conduction studies (NCS) and electromyography (EMG) can aid in differentiating the two conditions. It is possible to develop both conditions simultaneously, which poses unique challenges. We present a case of a woman who had diagnostic and clinical symptoms of both conditions, and discuss our management and the outcome of the patient.

## Introduction

Acute motor-sensory axonal neuropathy (AMSAN) is an uncommon, yet severe, variant of Guillain-Barré syndrome (GBS), accounting for less than 5%-10% of all GBS cases [1]. The pathogenesis in AMSAN is driven by axonal damage, which carries a worse prognosis compared to the more common demyelinating variants of GBS. This damage is mediated by anti-ganglioside antibodies, such as anti-GM1 and anti-GD1a, which target sodium channels on the nodes of Ranvier, and, in severe cases, the dorsal and ventral roots [2]. Axonal neuropathy can also be caused by nutritional deficiencies, particularly from thiamine (vitamin B1) deficiency, which is a sensorimotor polyneuropathy known as dry beriberi. The damage in this condition is caused by disruption of cellular energy metabolism [3].

AMSAN is an acute disorder that progresses over days, while dry beriberi typically develops subacutely over weeks [2,4]. Although both conditions are characterized by length-dependent (distal onset) motor and sensory neuropathy, they differ in several important aspects. Autonomic nervous system (ANS) involvement is more common and more severe in AMSAN [1]. Severe thiamine deficiency can present with other neurological complications, such as Wernicke's encephalopathy or Wernicke-Korsakoff syndrome [5]. Important differences also exist in the management and prognosis, as dry beriberi often has a favorable response to thiamine supplementation, while AMSAN requires immunomodulatory treatment with intravenous immunoglobulin (IVIG) or plasma exchange (PLEX), and carries a worse prognosis [1,5]. 

Nerve conduction studies (NCS) are essential in differentiating between axonal and demyelinating neuropathies. The hallmark of axonal damage is reduced amplitudes of compound muscle action potential (CMAP) and sensory nerve action potential (SNAP) [6]. Demyelinating neuropathy is characterized by slowed conduction, with NCS showing conduction block, temporal dispersion, prolonged distal latency, and reduced conduction velocity [7]. In axonal neuropathy, electromyography (EMG) can show denervation changes, including fibrillation potentials, positive sharp waves, and reduced motor unit recruitment [6].

AMSAN and dry beriberi have overlapping features on NCS and EMG, since they are both sensorimotor axonal neuropathies. However, AMSAN typically has more acute and more pronounced findings, while dry beriberi tends to be milder. Some studies have found that the sural sparing pattern on NCS is more common in AMSAN (30%) than in thiamine deficiency (8%) [8,9]. This pattern is characterized by upper extremity (UE) involvement with sparing of the sural nerve. 

The co-occurrence of AMSAN and dry beriberi should be promptly recognized and treated to improve the long-term outcome and prevent irreversible axonal degeneration. In this article, we describe the clinical and electrodiagnostic features of this rare occurrence and report the outcome of treatment. We also review the relevant literature.

## Case presentation

We present a 25-year-old Hispanic female with a pertinent medical history of obesity, recent vomiting related to a urinary tract infection (UTI), and chronic corticosteroid use for severe allergic reaction to laundry detergent, who was treated at our hospital for rapidly progressive sensorimotor neuropathy, complicated by respiratory failure.

The patient was initially admitted to the general medical ward for a UTI, complicated by pyelonephritis and septicemia, managed with intravenous (IV) ceftriaxone. Nephrolithiasis requiring nephrostomy placement prolonged her hospitalization, though she remained neurologically intact and tolerated a regular diet throughout. On admission day 30, she started developing difficulty ambulating to the bathroom of the hospital room and complained of numbness in her hands and feet. On admission day 37, and seven days after the start of neurological symptoms, her weakness worsened to being unable to stand, and she developed blurred vision. Neurological examination showed a normal mental status; tachycardia (124 beats per minute); Medical Research Council (MRC) strength grade of 4/5 in the UEs and 2/5 in the lower extremities (LEs); diffusely reduced deep tendon reflexes; and distal symmetric UE and LE light touch and proprioception impairment. She exhibited a bidirectional, gaze-evoked, non-fatigable horizontal jerky nystagmus, with full range of extraocular movements.

Pertinent findings in the cerebrospinal fluid (CSF) analysis include a protein of 154 mg/dL and a white blood cell (WBC) count of 1/μL. As indicated in Table 1, the patient also demonstrated an elevated CSF IgG (immunoglobulin G) index (0.82) and increased IgG concentration (15.9 mg/dL), along with two oligoclonal bands, suggesting mild intrathecal immune activation. She was also deficient in thiamine (B1), pyridoxine (B6), and copper, indicating a concurrent nutritional deficiency that may have contributed to her neuropathy. These findings point to a possible overlap of inflammatory and metabolic etiologies.

**Table 1:** Important testing results CSF, cerebrospinal fluid; IgG, immunoglobulin G

Labs	Date Obtained Since Neurological Symptom Onset	Values (Normal Levels)
CSF glucose	Day 8	77 mg/dL
CSF IgG index	Day 8, reported on day 11	0.82 ratio (0.28 - 0.66)
IgG value	Day 8, reported on day 11	15.9 mg/dL (0.0-6.0)
Oligoclonal bands	Day 8, reported on day 10	2 (0-1)
B1	Day 9, reported on day 14	29 nmol/L (70-180)
B6	Day 10	18.8 nmol/L (20-125)
Zinc	Day 22	84.8 ug/dL (60-120)
B12	Day 10	521 pg/mL (240-930)
Folate	Day 10	3.7 ng/mL (3-20)
Repeated B1 (after supplementation)	Day 17, reported on day 22	277 nmol/L (70-180)
Copper	Day 22	36.5 ug/dL (80-155)
Repeated B6 (after supplementation)	Day 38	71 nmol/L (20-125)

**Figure 1:**
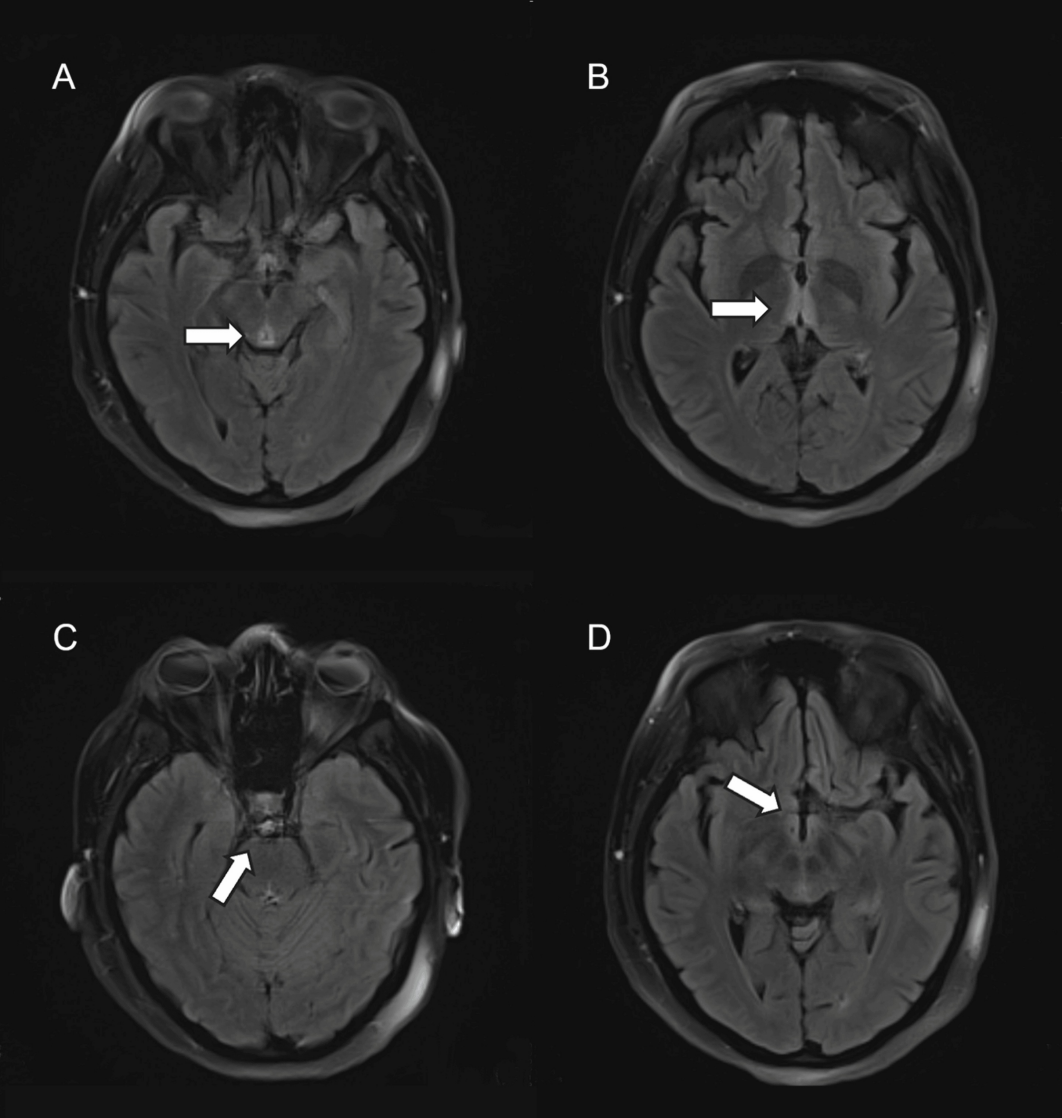
FLAIR MRI images showing hyperintense lesions (arrows) (A) Periaqueductal grey matter; (B) Dorsomedial thalamus; (C) Basis pontis; (D) Mammillary bodies FLAIR, fluid attenuation inversion recovery; MRI, magnetic resonance imaging

As seen in Figure 1, magnetic resonance imaging (MRI) of the brain and neuroaxis showed features suggestive of Wernicke's encephalopathy. NCS showed features of severe sensorimotor axonal neuropathy (Table 2). Needle EMG, including facial testing, was not performed due to the patient’s clinical instability during the diagnostic window. While EMG may have further characterized the extent of motor involvement, the NCS findings and clinical presentation were sufficient to support the diagnosis. She was treated with 500 mg of thiamine supplementation intravenously three times daily (TID) for five days, followed by 100 mg daily, and was simultaneously started on IVIG 2 g/kg over five days, but no significant improvement was achieved.

**Table 2:** Nerve conduction studies results CMAP, compound muscle action potential; SNAP, sensory nerve action potential; L, left; R, right; NR, no measurable response despite supramaximal stimulation; N, normal

Nerve Stimulated	Stimulation Site	Latency (ms)	CMAP Amplitude (mV)	SNAP Amplitude (µV)	Conduction Velocity (m/s)	Conduction Distance (cm)	Interpretation
Median L/R (motor)	Wrist	3.6/3.4 (Distal N: <4.0)	0.7/1.7 (N: >6.0)	-	54/63 (N: >48)	-	Reduced CMAP amplitude with preserved distal latency and normal conduction velocity is consistent with axonal motor neuropathy
Median L/R (motor)	Elbow	7.7/6.6 (Proximal)	0.6/1.6 (N: >6.0)	-	54/63 (N: >48)	-	Reduced CMAP amplitude with preserved distal latency and normal conduction velocity is consistent with axonal motor neuropathy
Peroneal L/R (motor)	Fibular head	NR/NR (N: <6.6)	NR/NR (N: >2)	-	NR/NR (N: >41)	-	Absent CMAP amplitude indicates severe axonal neuropathy
Median L/R (sensory)	Second digit	NR/NR (N: <3.6)	-	NR/NR (N: >15)	-	14/14	Absent SNAP indicates severe axonal neuropathy
Sural L/R (sensory)	Lateral malleolus	NR/NR (N: <4.5)	-	NR/NR (N: >6)	-	14/14	Absent SNAP indicates severe axonal neuropathy

On admission day 47, and 17 days following the onset of neurological symptoms, she developed respiratory difficulty and was started on non-invasive ventilation, followed by intubation three days later due to deteriorating mental status. She also developed autonomic dysfunction, as she required vasopressors for hypotension, and developed urinary retention and paralytic ileus. At this point, her UE involvement became more pronounced, as her strength was 1/5 based on the MRC score and 2/5 in the LE. We then administered four sessions of PLEX with no improvement. She remained intubated and was discharged to a long-term acute care facility with vitamins B1 and B6 supplementation, as well as cetirizine (after consultation with Allergy and Immunology). She was eventually weaned off the ventilator.

At her five-month neurology follow-up, the patient was alert with normal mental status and her nystagmus had resolved. While still bedridden, she showed motor improvement with UE strength of 4/5 and LE strength of 3/5 (MRC scale). The Achilles reflex improved, while other deep tendon reflexes remained diminished. Sensory deficits had also improved.

## Discussion

As AMSAN and dry beriberi can both cause sensorimotor axonal neuropathy, careful differentiation between them is essential to prevent misdiagnosis. Our patient likely had a combined presentation, which highlights unique aspects in the diagnosis and management.

Our patient presented with acute symptoms and had a pronounced reduction in the CMAP and SNAP amplitudes, which argues for AMSAN, but also had clinical and MRI features of Wernicke’s encephalopathy, which makes the concomitance of dry beriberi plausible. 

As shown in Table 3, we identified seven articles reporting eleven patients with thiamine deficiency initially misdiagnosed as AMSAN [10-16]. Only two cases have reported the concomitance of both conditions [17,18].

**Table 3:** Clinical features of previously reported cases *Refers to the patient reported in this article. All patients had low thiamine except #3 and #8. Patient #10 received thiamine supplementation; therefore, testing was deferred. Weakness is classified as severe if the MRC is 0-3, moderate if 4 to 4+, and mild if greater than 4+. CSF, cerebrospinal fluid; MRI, magnetic resonance imaging; EMG, electromyography; NCV, nerve conduction velocity; MRC, Medical Research Council

Author	Patient Number	Age and Gender	Progression	Weakness Severity	Cranial Nerve Involvement	Autonomic Involvement	Respiratory Involvement	Testing	Prognosis
Salazar et al.*	#0	25, female	25 days	Severe	Blurred vision and nystagmus	Tachycardia	Intubation	CSF with albuminocytologic dissociation, MRI brain showed signs of Wernicke’s Encephalopathy, EMG/NCV showed axonal sensorimotor neuropathy	Minimal recovery
Koike et al. [10]	#1	46, male	28 days	Severe	Ophthalmoplegia	Urinary retention and obstipation	Not reported	EMG/NCV showed axonal sensorimotor neuropathy	Full recovery
	#2	33, male	14 days	Severe	None	Not reported	Not reported	EMG/NCV showed axonal sensorimotor neuropathy	Not reported
Murphy et al. [11]	#3	44, male	21 days	Severe	None	Miosi	None	CSF with albuminocytologic dissociation, MRI consistent with central pontine myelinolysis, EMG/NCV showed axonal sensorimotor neuropathy	Moderate improvement
Benameur and Clarke [12]	#4	24, female	14 days	Severe	Blurry vision	Urinary retention	Not reported	EMG/NCV showed axonal sensorimotor neuropathy	Not reported
	#5	40, female	5 days	Severe	None	Disconjugate gaze	Not reported	MRI brain showed signs of Wernicke’s Encephalopathy, EMG/NCV showed axonal sensorimotor neuropathy	Not reported
	#6	48, female	30 days	No weakness	Not reported	Thermal dysregulation	Not reported	EMG/NCV showed axonal sensorimotor neuropathy	Not reported
	#7	43, female	Not reported	Severe	Nystagmus	Not reported	Not reported	MRI brain showed signs of Wernicke’s Encephalopathy EMG/NCV showed axonal sensorimotor neuropathy	Not reported
Shible et al. [13]	#8	56, female	30 days	Not reported	Not reported	Hypotension	Intubation	CSF with albuminocytologic dissociation, MRI brain showed signs of Wernicke’s Encephalopathy, only EMG done showing signs of motor neuropathy	Marked improvement
Faigle et al. [14]	#9	62, male	14 days	Mild	Abducens palsy	Tachycardia	Not reported	Elevated CSF protein (125 mg/dL), MRI brain showed signs of Wernicke’s Encephalopathy EMG/NCV showed axonal sensorimotor neuropathy	Not sufficiently reported
Gan et al. [15]	#10	26, female	30 days	Severe	Nystagmus and dysarthria	Not reported	Not reported	CSF with albuminocytologic dissociation, MRI brain showed signs of Wernicke’s Encephalopathy, EMG/NCV showed axonal sensorimotor neuropathy	Moderate improvement
Riahi et al. [16]	#11	14, female	30 days	Moderate	Facial weakness, dysphonia, dysphagia	Not reported	Not reported	EMG/NCV showed axonal sensorimotor neuropathy	Full recovery
Ghonim et al. [17]	#12	31, female	21 days	Not reported	Not reported	Tachycardia	Not reported	EMG/NCV showed axonal sensorimotor neuropathy	Not sufficiently reported
Daigle et al. [18]	#13	62, female	22 days	Severe	Not reported	Not reported	Not reported	CSF with albuminocytologic dissociation, MRI brain showed breast cancer metastasis EMG/NCV showed axonal neuropathy	Near-complete recovery

Our patient had a recent past medical history of significant vomiting, which could have contributed to thiamine deficiency. Of the 13 patients in prior studies, two had a recent history of vomiting [12,15], and 11 reported a history of either gastrectomy (n = 3), dietary insufficiency (n = 6), celiac disease (n = 1), or breast cancer (n = 1) [10,11,13,14,16-18].

The primary symptoms in our patient were rapidly progressive ascending weakness and sensory loss. Features in our patient that are shared with the majority of documented cases include severe weakness, determined by the MRC scale, as well as signs of encephalopathy. Our patient’s development of nystagmus and blurred vision points to cranial nerve dysfunction, which was identified in only six patients [10,12,14-16]. Although five patients developed autonomic dysfunction characterized by either urinary or fecal retention, ophthalmic abnormalities, or impaired thermoregulation, our patient had extensive autonomic dysfunction characterized by both tachycardia and hypotension, in addition to bowel and bladder deficits [10-12]. Only three patients had either tachycardia or hypotension [13,14,17]. A unique feature of our patient was her degree of respiratory involvement; she was intubated for two months. Intubation occurred in only one other patient, who was intubated for just three days [13].

All patients were identified as having sensorimotor axonal neuropathies through either NCS or EMG. Additionally, almost all patients were identified as having low serum levels prior to treatment with thiamine. Three patients were started on prophylactic thiamine, and pre-treatment levels were not checked [11,13,15]. Only four patients had either CSF findings of albuminocytologic dissociation [11,18] or cranial MRI consistent with Wernicke's encephalopathy [12]; meanwhile, three patients had abnormalities in both diagnostic studies [13-15]. Concomitant MRI findings of Wernicke's encephalopathy and CSF findings of albuminocytologic dissociation pose a challenge to diagnosis, as thiamine deficiency does not cause CSF abnormalities, and AMSAN does not cause Wernicke's encephalopathy. The differences in response to treatment and outcome are what separate our patient from these cases with similar diagnostic findings.

All but one patient were treated with thiamine (the breast cancer patient was treated with IVIG only) [18]. Interestingly, only one patient of the three with CSF abnormalities and MRI findings consistent with Wernicke's encephalopathy was treated with both IVIG and thiamine [15]. Our patient was treated with 500 mg IV thiamine TID for five days, followed by 100 mg daily, as well as IVIG 2 g/kg over five days to cover for concomitant thiamine deficiency and AMSAN. 

All patients whose condition post-treatment was described showed at least moderate improvement and no worsening of symptoms [10,11,13,16,18]. On the contrary, our patient continued to deteriorate rapidly, even after administration of thiamine (for dry beriberi) and IVIG (for AMSAN), as she developed autonomic dysfunction and was intubated. Five months after discharge, she was no longer intubated and regained most of her UE strength; however, she remained bedridden. The rapid progression of neurological damage and slow recovery was likely due to concomitant AMSAN. 

Although thiamine deficiency is described as rare in developed countries, it has a prevalence of about 15%-30% in obese patients [19]. Reasons include a diet high in carbohydrates and a high incidence of bariatric surgeries, which limit the absorption of thiamine. In our patient, it is possible that her obesity played a role in low thiamine levels and the development of Wernicke's encephalopathy, but likely, the primary etiology of her neuropathy was a variant of GBS. 

## Conclusions

This report aims to emphasize the importance of identifying both thiamine deficiency and AMSAN as possible etiologies for sensorimotor neuropathy. Obtaining pertinent patient history is the first step in differentiating AMSAN from thiamine deficiency. Evidence of dietary insufficiency, history of gastrectomy, or chronic vomiting will likely favor a diagnosis of thiamine deficiency; meanwhile, a history of viral infection may signal an autoimmune cause, thereby favoring the diagnosis of AMSAN. The sural sparing phenomenon in NCS is more indicative of AMSAN than thiamine deficiency. MRI imaging can be used to see if there is evidence of Wernicke's encephalopathy, which would point to thiamine deficiency. In cases of concomitant presentations, as in our case, it is imperative to recognize both and manage accordingly to prevent the progression of the paralysis.

Limitations of our study include the absence of needle EMG, which could have strengthened diagnostic certainty, ruled out differentials such as critical illness myopathy, and assessed facial muscle involvement. Additionally, while mild CSF abnormalities (e.g., elevated IgG index and oligoclonal bands) were present, they did not strongly support a definitive inflammatory or immune-mediated process. Although the five-month follow-up demonstrated meaningful clinical improvement, longer-term follow-up would provide further insight into the recovery trajectory and residual deficits.
